# Metastatic Upper Thoracic Intramedullary Spinal Cord Tumor of Ovarian Adenocarcinoma: A Rare Case Report and Literature Review

**DOI:** 10.1002/cnr2.70013

**Published:** 2024-10-16

**Authors:** Vahid Heidari, Reza Mollahoseini, Navid Golchin, Hadi Karimi, Mohammad Hoseini, Benyamin Kazemi, Elmira Zolfeghari, Hemin Ashayeri Ahmadabad

**Affiliations:** ^1^ Department of Neurosurgery, School of Medicine, Firoozgar Hospital Iran University of Medical Sciences Tehran Iran; ^2^ Department of Neurology Inkosi Albert Luthuli Central Hospital, University of KwaZulu‐Natal, Nelson, Mandela School of Medicine Durban South Africa; ^3^ Department of Pathology, School of Medicine, Firoozgar Hospital Iran University of Medical Sciences Tehran Iran; ^4^ Islamic Azad University of Medical Science Tehran Iran

**Keywords:** case report, intramedullary spinal cord metastasis, neoadjuvant chemotherapy, ovarian adenocarcinoma, rare metastatic condition, surgery

## Abstract

**Background:**

Intramedullary spinal cord metastasis (ISCM) is uncommon and usually occurs in advanced malignancies. Effective management methods are not clearly defined, and the outcomes of current treatments vary. Currently, there is no universal strategy for managing patients with intramedullary spinal metastases.

**Case:**

To the best of our knowledge, we present a case of ovarian adenocarcinoma that was managed surgically in a 70‐year‐old woman with metastasis to the upper thoracic spinal cord, and we assess pertinent literature and deliberate management approaches.

**Conclusions:**

Progression in early diagnosis and advanced therapeutic methods for ISCMs have contributed to the increased incidence and prevalence of this condition. There is no established consensus regarding the definitive patient management methods. Consequently, we offer multidisciplinary management with individualization based on the patient's functional status, requirement for a definitive diagnosis for potential additional adjuvant therapies, and assessment of the extent of systemic disease, which can influence the desired quality of life and survival duration.

## 1 Introduction

Recent studies indicate that there is an expected increase in the global cancer load over the next few years [1]. Previous studies showed that in 2020, approximately 19.3 million new cancer cases were diagnosed and approximately 10 million died during this year [2]. Scientists have predicted that the incidence of cancer diseases is expected to increase to about 28.4 and 34 million in 2040 and 2070, respectively [1, 2]. In Iran, there is an estimated 42.6% increase in incidence from 112 000 cases in 2016 to 160 000 in 2025 [3]. Most cancer‐related deaths occur due to metastasis [4]. The liver, lung, and skeletal system are the most common sites of metastasis, within the skeletal system, the spinal column is the most common site of metastasis [5]. Although spinal metastases in vertebrae and the extradural space of the spinal cord are common, intramedullary spinal cord metastasis (ISCMs) is a rare condition that occurs in less than 5% of all spinal metastases and 4.2%–8.5% of central nervous system (CNS) metastases [6]. The prevalence of ISCM is increasing due to the widespread use of advanced diagnostic instruments and the increasing survival of cancer patients [7]. Among different primary tumors, breast, lung, and prostate cancers are the most common primary cancers, which metastasize to the spinal cord [8]. Less commonly, ovarian cancer is a primary disease, that metastasizes to the spinal cord [9]. Metastasis of ovarian cancer in the CNS ranges between 0.29% and 11.6%. Advanced management of ovarian cancer results in increased patient survival, which may be one of the reasons for metastasis to the CNS [10]. While there is no consensus in the literature about the most common level of ISCM, the thoracic and cervical regions are frequently cited as sites of metastasis [11, 12]. Previous studies have indicated that ISCM occurs through various mechanisms, such as hematogenous invasion through the Batson vein and the arterial system, drop metastasis from leptomeningeal lesions, perineural invasion, and direct invasion from the epidural space through the nerve roots [11, 13, 14]. The symptoms of ISCMs vary depending on the site [12]. The most common presentations of the patients included pain (83%–95%), weakness (60%–85%), sensory dysfunction (40%–90%), and less common dysfunction of the autonomic system (40%–57%) [9]. ISCMs are characterized by highly malignant neoplasms, rapid neurological deficits, and rapid progression. One typical feature of ISCM is the rapidly worsening of neurological impairment, distinguishing it from other primary intramedullary neoplasms such as glioma, representing a significant cause of myelopathy progression over a short time [11]. Approximately half of the patients experience rapid deterioration of neurological deficits and progression to hemisection of the spinal cord [10], which may be caused by the rapid progression of ISCMs. In some patients, the lesions may be asymptomatic, incidentally diagnosed, or may go unnoticed and be detected at autopsy [12]. Due to the rare incidence of ovarian carcinomas and limited available information in the literature, there is a lack of well‐established diagnostic strategies, and management protocols are less defined. In treated ovarian carcinoma, routine follow‐up involves a contrast computed tomography (CT) scan of the chest and abdomen as the omentum, viscera, and diaphragm, along with CA‐125 levels, which can be misleading [15, 16]. Additionally, utilizing lumbar puncture (LP) and magnetic resonance imaging (MRI), which are the gold standards for diagnosis, is helpful for early detection [11]. Based on the previous studies, treatment for ISCM remains non‐standard, and the neurological findings of patients are the principles guiding treatment methods [12]. There are various treatment methods, including radiotherapy, chemotherapy, surgery, radiosurgery, embolization of tumor vessels, and also multimodality treatment [12, 17]. The essential goal of treatment is to manage the signs and symptoms and improve neurologic outcomes. Despite improvements in therapeutic methods, there is a poor prognosis and low survival in ISCM patients [12].

In this case report, we introduce a 70‐year‐old woman who underwent ovarian carcinoma treatment 3 years ago in 2017 and was thought to be free of disease but recently presented with spinal cord metastasis, which is a rare entity. In addition, she presented with a progressive neurological deficit while being admitted. She was admitted to the Firoozgar Hospital in Tehran, Iran, in 2020 with ISCM. As we can see, the prognosis of these patients is highly dependent on their neurological status in the admission time. Therefore, rapid evaluation of neurological status is vital, since missing time may impose a significant burden on patient morbidity and mortality. Early diagnosis of ISCM, can significantly improve the outcomes. Here, we also review articles related to spinal cord metastasis from ovarian cancer and discuss the diagnosis, therapeutic methods, outcome, and prognosis.

## 2 Case Report

### 2.1 Case Presentation

A 70‐year‐old woman arrived at the Firoozgar Hospital in Tehran/Iran in 2020, with a history of Stage IV papillary serous carcinoma in the left ovary, peritoneum, and omentum, that underwent surgical removal of the uterus and cervix (total abdominal hysterectomy), both the ovaries and fallopian tubes (bilateral salpingo‐oophorectomy), and omentum (omentectomy). Additionally, the patient underwent resection of two hepatic masses simultaneously. All surgeries were performed approximately 3 years ago. Furthermore, at the time of surgery, her condition was not identified as metastatic disease, according to the information provided by the patient's family.

The patient was treated with palliative chemotherapy and shifted to the next line of chemotherapy on progression. The patient's medical history was positive for a brain aneurysm clipping on the anterior communicating artery 20 years ago, which had no complications including cerebrospinal fluid (CSF) leak, visual field defect, or ischemic stroke. One month before admission, she developed an insidious onset of paraparesis. This was followed by severe paraparesis with lower extremities force 1/5 (proximal and distal) affecting her mobility and dexterity, and impaired sensation in the upper thoracic sensory level (paraesthesia) that progressed to the lower limb. She also developed bowel and urinary sphincter incontinence in the days preceding her admission to the hospital.

### 2.2 Diagnostic Imaging

**FIGURE 1:**
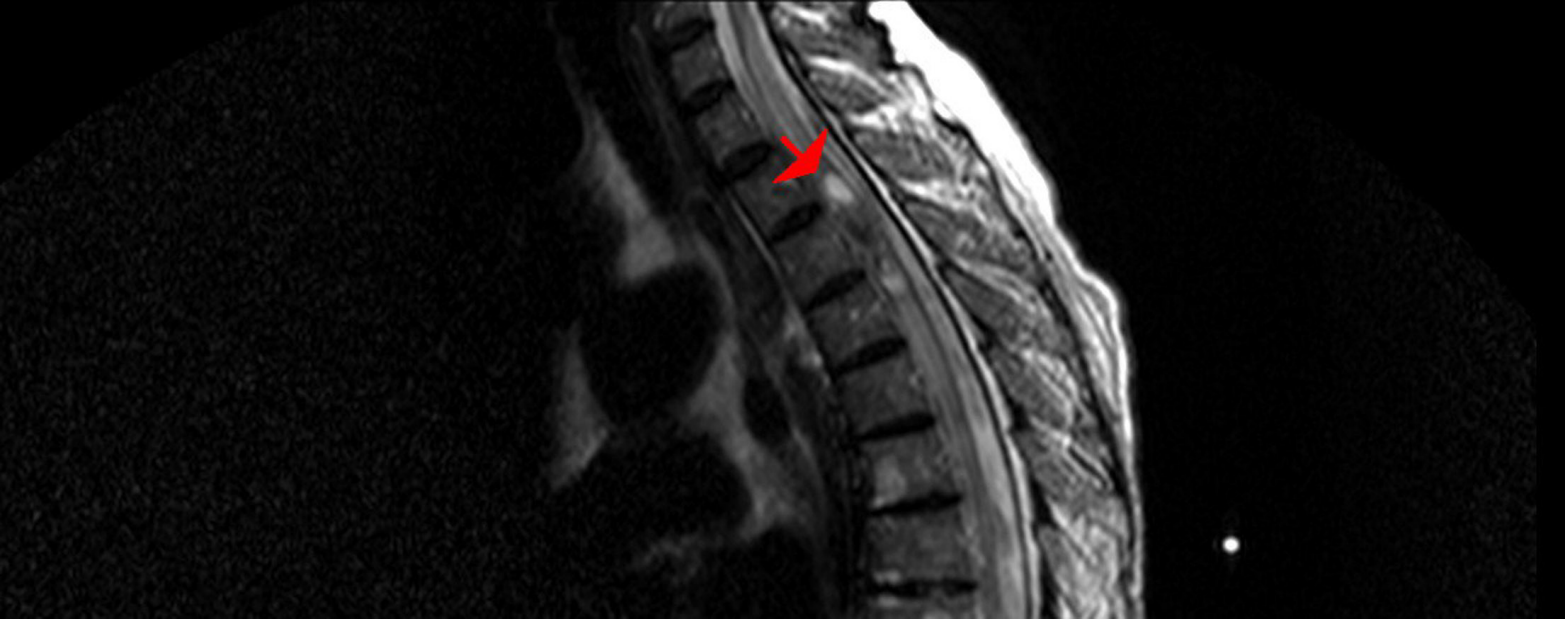
Sagittal T2 spinal MRI shows an intradural intramedullary tumor at T2, T3 level (hyposignal lesion with 2 hypersignal portion and proximal syrinx formation).

**FIGURE 2:**
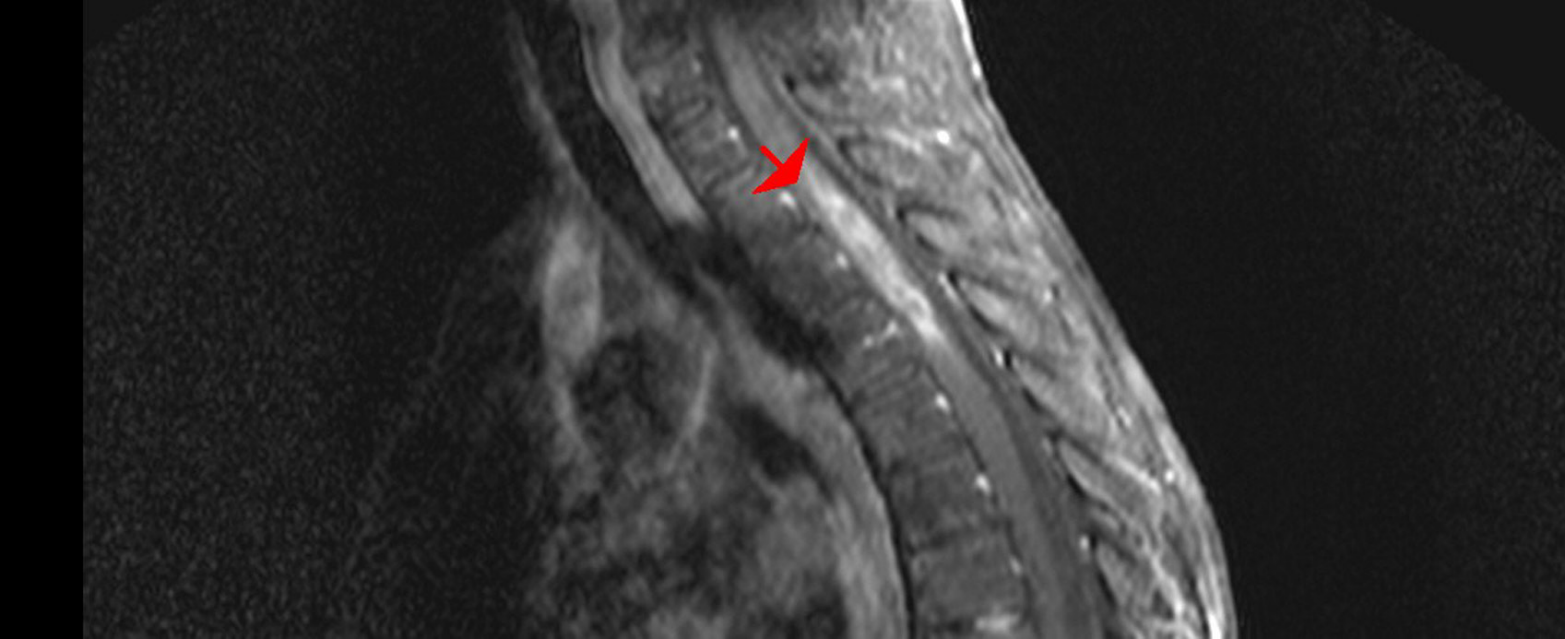
Thoracic MRI post GAD. Sagittal T1 Gd + spinal MRI shows an intradural intramedullary tumor at T2, T3 level (hyper signal lesion or avidenhancement).

An MRI of the spinal cord demonstrates a relatively poor circumscribed intramedullary lesion centered in the thoracic cord at the T2/T4 disc level and post‐contrast heterogeneous enhancement with gadolinium and cord edema from C7 to T8 (Figures 1 and 2). Based on her medical history and MRI findings, these raised concerns for spinal cord primary malignancy or metastasis.

### 2.3 Surgical Intervention

The patient underwent laminectomies at the T1, T2, and T4 levels followed by microscopic resection of the ISCM. A midline vertical myelotomy was conducted in the posterior portion of the spinal cord. The tumor was centrally located and highly vascular and exhibited a heterogeneous nature, featuring both soft and relatively hard components. Notably, it exhibited varied coloration, with some portions appearing grayish while others had a brownish hue. The tumor was removed piecemeal, with necrotic parts suctioned and the other dissected. No complications occurred during the surgery, and the patient tolerated the surgery procedure fine without new neurological impairment through the postoperative period. Unfortunately, there was no improvement in her ability to walk. Additionally, there was no improvement in motor and sensory deficits of the limbs, nor in her bowel and bladder incontinence.

### 2.4 Outcome

The patient was discharged 7 days post‐surgery with a follow‐up appointment scheduled for 10 days later. Also, she was planning on receiving adjuvant chemo/radiotherapy later. Following subsequent follow‐up, it was observed that. 3 weeks after surgery, the patient succumbed to respiratory and circulatory failure. Her family declined to authorize an autopsy.

### 2.5 Histopathology

**FIGURE 3:**
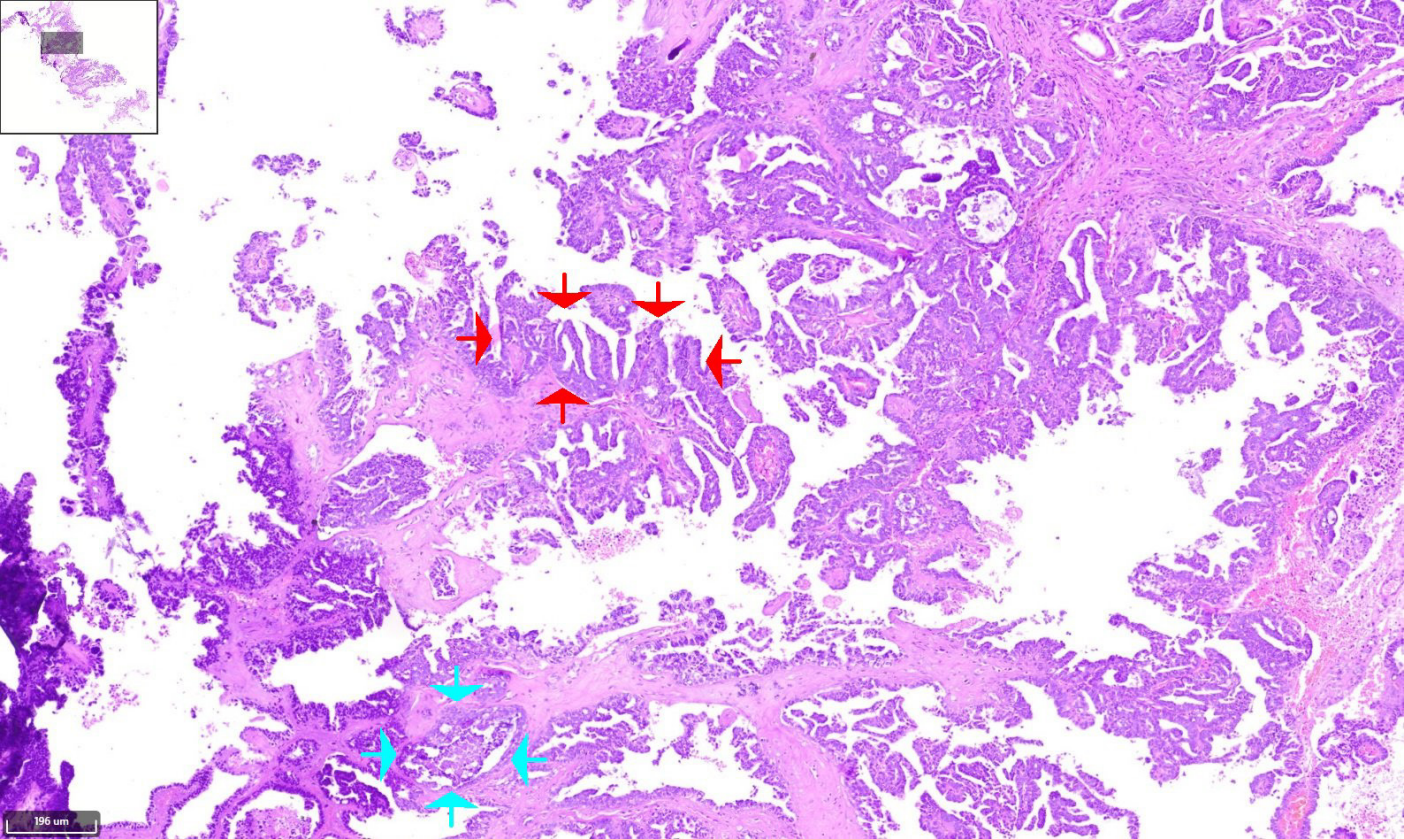
Hematoxylin and eosin (H&E) stained of serous papillary carcinoma of upper thoracic intramedullary spinal mass shows prominent papillary (red arrows), micropapillary, and cribriform (blue arrows) structures of large atypical cells with infiltrative border and desmoplastic background. Papillary pattern reveal branching and architectural complexity. (×10).

**FIGURE 4:**
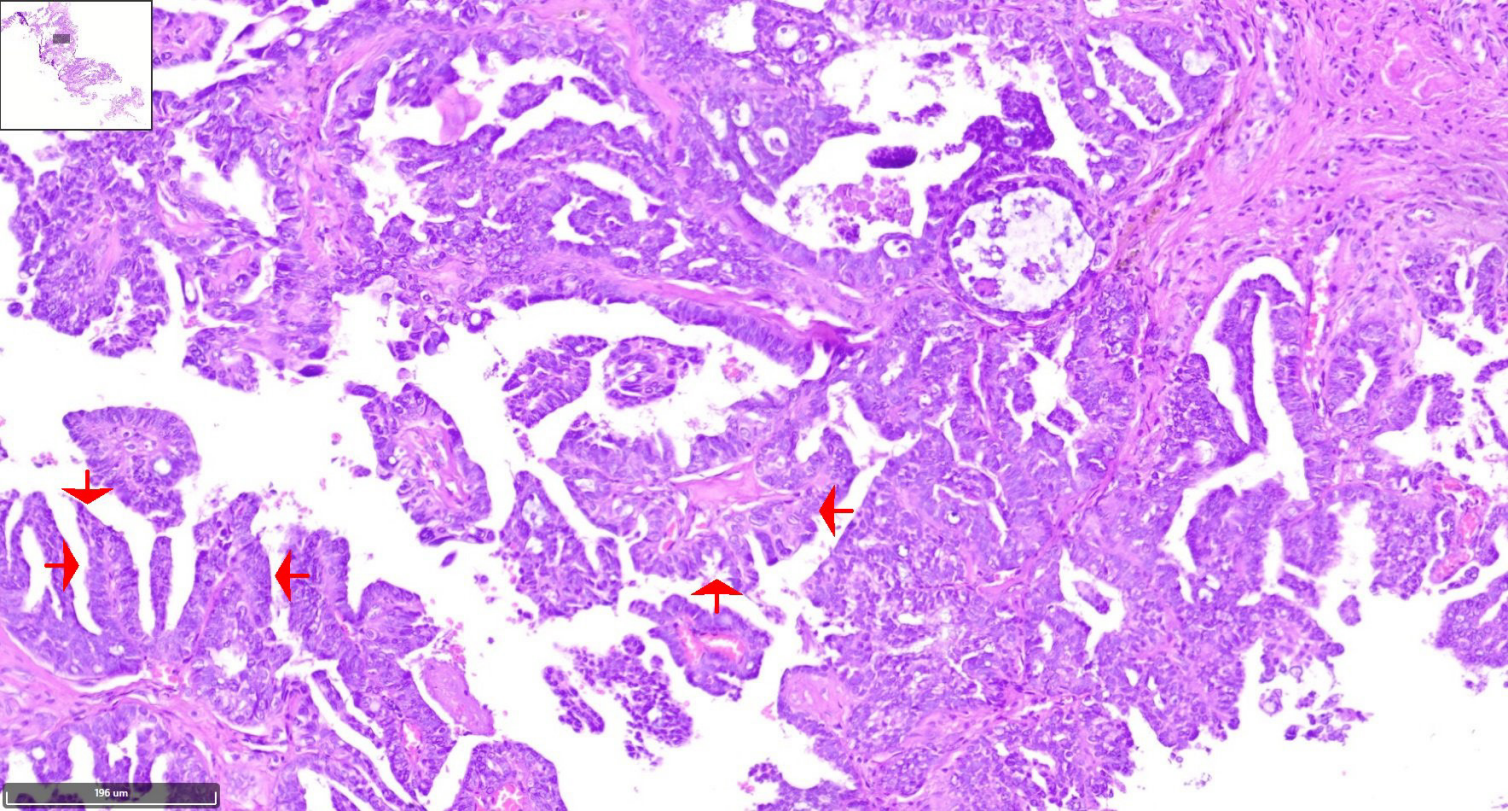
H&E stained of low‐power image adjusted to a high‐power image of the tumoral papillary projection reveals papillae formations lined by large atypical cells with high nucleus‐to‐cytoplasmic (N:C) ratio with extensive necrosis in background. The tumoral cells have irregular hyperchromatic nuclei, prominent nucleoli, and moderate amount of eosinophilic cytoplasm. (×10) (red arrows).

**FIGURE 5:**
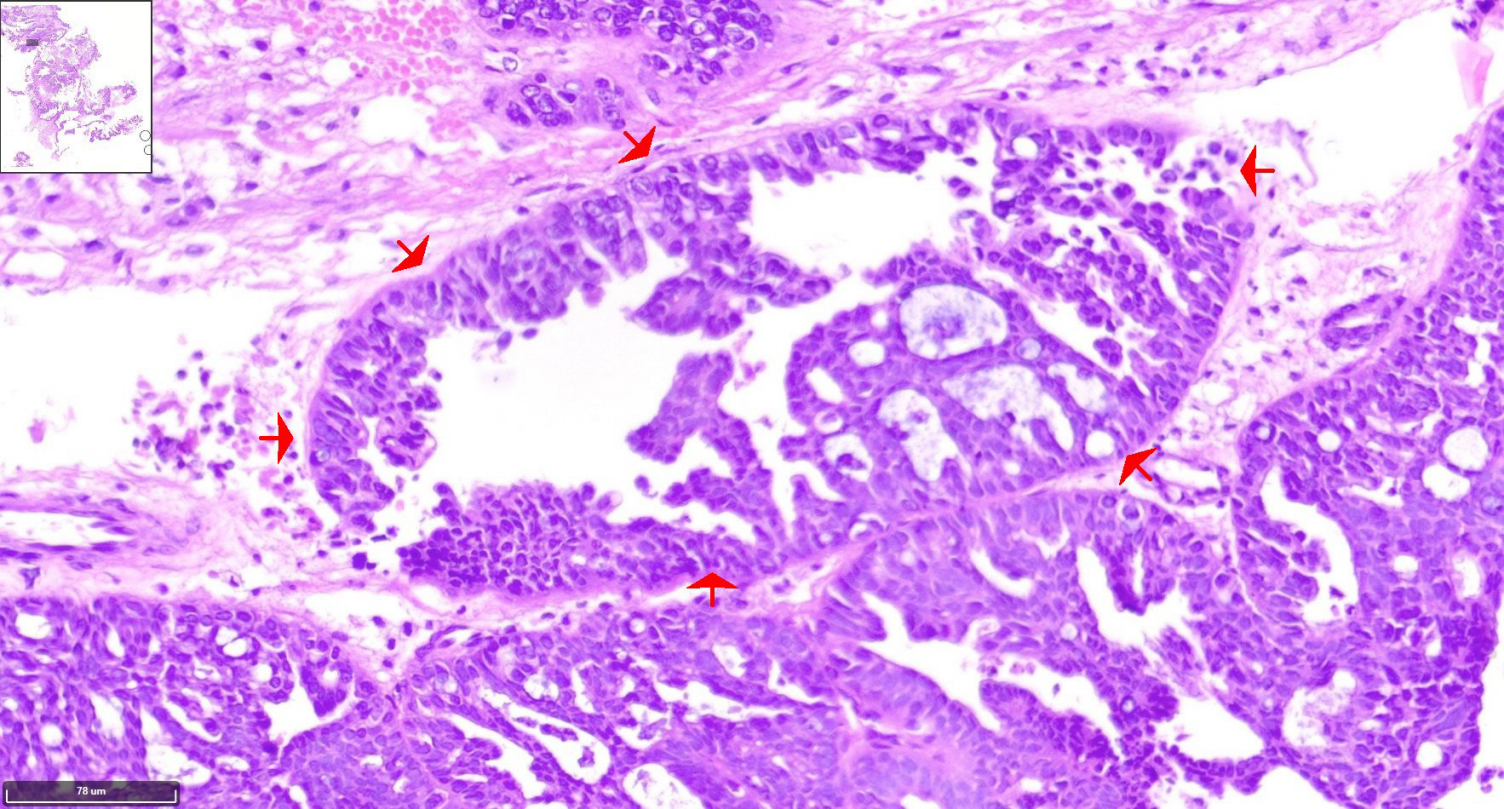
The H&E stained tissue section reveals cribriform pattern of tumor with nuclear pleomorphism, which is common in serous papillary carcinoma (×20) (red arrows).

**FIGURE 6:**
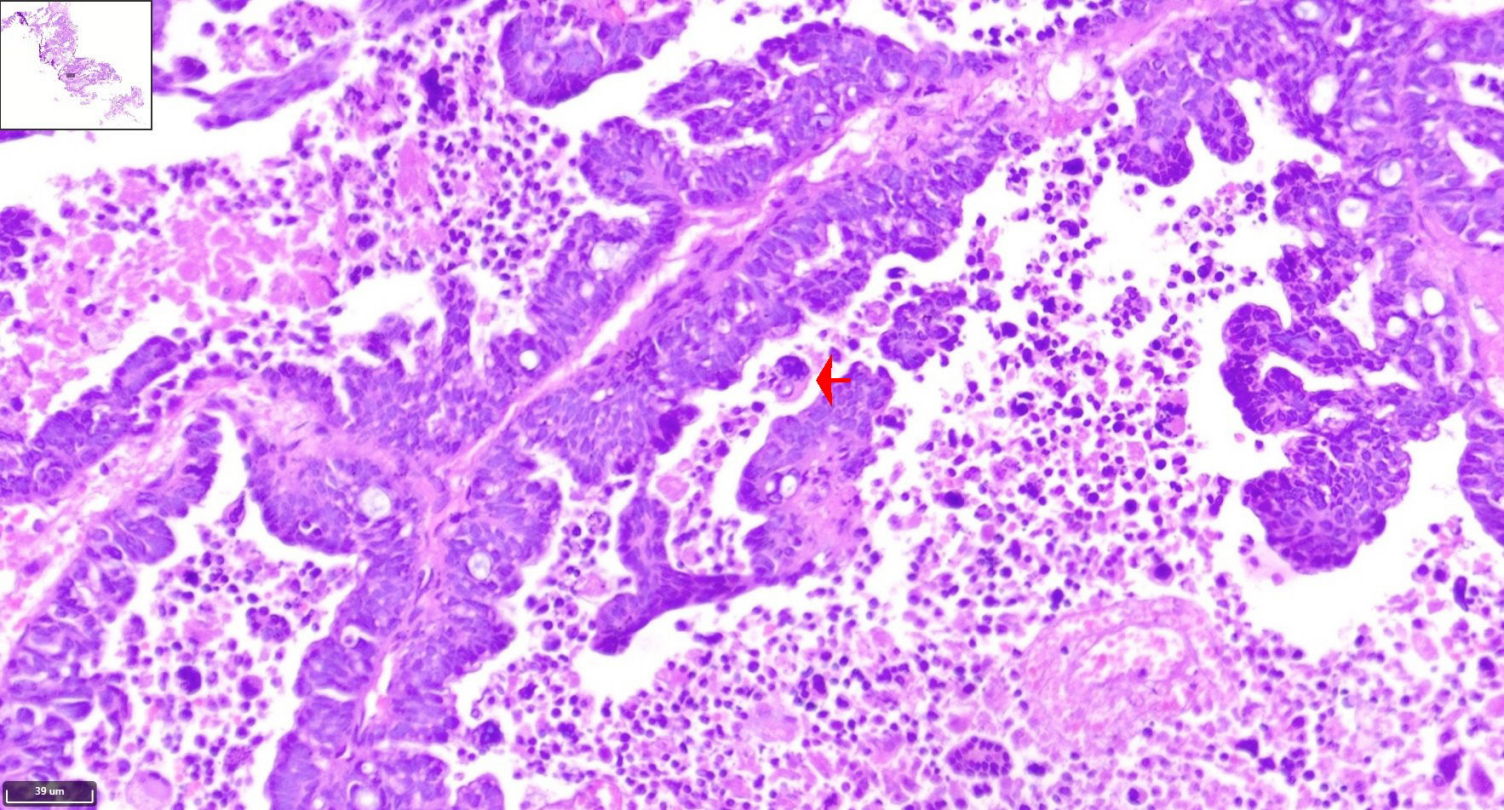
Papillary serous ovarian adenocarcinoma, frequent mitotic figure, and atypical mitosis (black arrow) are noted (H&E staining, ×60) (red arrow).

Histopathological analysis showed extensive papillary architecture (Figures 3, 4, 5, 6). There was papillary branching, glandular, and cribriform patterns, growing in solid. Significant nuclear atypia such as nuclear pleomorphism (>3× variation in size), large, bizarre, multinucleated nucleus forms were noted. Also, prominent large and eosinophilic nucleolus and eosinophilic cytoplasm were seen. Areas of necrosis were frequent. A high mitotic index (≥12 mitotic figures per 10 high‐power fields) was measured. Immunohistochemistry (IHC) analysis was positive for PAX8 and WT1 proteins, which was indicative of papillary serous ovarian adenocarcinoma. The histological and immunohistochemical characteristics of the lesion were in consistent with a metastatic papillary serous ovarian adenocarcinoma.

## 3 Discussion

The ISCMs are an extremely rare condition, with up to 2.1% reported prevalence in all cancers. Additionally, 2.1% of all ISCMs are from primary ovarian cancer [16]. An important site of metastasis from solid tumors is the intramedullary spinal cord [18]. While the mechanism of distant metastasis of ovarian cancer remains unclear, understanding the pathophysiology of metastasis may help for explanation of patients' symptoms. Some studies suggest that the mechanism of metastasis in ovarian cancer differs from others. They indicate that serous ovarian carcinoma progresses significantly in the peritoneal cavity, while less commonly metastasizing [9]. Some suggest that hematogenous dissemination is a basic contributor to distant metastasis. Another hypothesis suggests that in advanced stages, single cells or a cluster of tumor cells detach and drop, then they disseminate through the peritoneal fluid into the peritoneum and omentum [9, 15, 16]. In addition to the spinal cord, distant metastases to the brain, liver, and other regions are reported, while the clinical predictors are obscure. Some studies showed a correlation between distant metastasis with p53 mutations, genomic instability, staging, and tumor vascularity [9]. The clinical presentations of ISCMs vary widely, sometimes asymptomatic or nonspecific. Most patients show radicular pain, weakness, sensory loss, and autonomic impairment, depending on the level of cord involvement [19]. Our case was admitted with diminished lower extremities strength, the sensory deficit at the upper thoracic level, which progressed to the lower limbs, and bladder and bowel incontinence.

Formerly, myelography was the diagnostic tool used when the MRI was not available. Myelography is associated with a high rate of false‐negative results [10]. Nowadays, several diagnostic tools aid in the diagnosis of ISCMs. For example, LP is presented as a diagnostic tool. Although LP usually does not show increased pressure of CSF, it can help in document hyperprotidorrhachia without cell alteration [11]. Another diagnostic instrument is MRI of the spine, which is the gold standard diagnostic tool [11, 19]. It usually shows a well‐circumscribed lesion with surrounding edema in a T2‐weighted image. The lesion may appear T2‐hyperintense, T1‐iso, or hypo‐intense, often homogenous and nodular, or less commonly heterogeneous with ring enhancement [11, 20]. Additionally, an urgent MRI is indicated for patients with a new or advancing neurological impairment [8]. In some patients, when MRI with and without gadolinium is not applicable, a CT scan can help diagnose ISCM [11]. Identifying the presence of calcification within the tumor can be aided by CT myelography [20]. Additionally, routinely following up on treated patients with contrast CT scan along with CA‐125 serum level can help in the early diagnosis of ISCMs [15, 16].

If untreated, neurological impairment rapidly worsens and progresses to a hemisection syndrome of the spinal cord or results in cord transection in nearly half of patients [16, 21]. Intramedullary spinal tumor treatment is controversial. Due to limited knowledge in determining the prognosis for patients with spinal cord metastasis, oncologists, and neurosurgeons face challenges in selecting the most beneficial method of treatment [18]. Various management methods depend on the patient's functional state, the definitive diagnostic tool to guide treatment options, and the extent of systemic disorder that impacts quality of life and survival time [9, 16, 22]. One treatment method is surgery, which aims to achieve cytoreduction, histological diagnosis, and relief of neurodificits [16]. For spinal cord cancers, complete surgical resection is the treatment of choice and can be curative for well‐defined border tumors [20]. Surgery is indicated in patients suffering from severe pain, neurological impairment, and spinal instability [19]. Another treatment method is systemic steroids, which can help diminish vasogenic edema associated with tumors, potentially relieving the patient's symptoms. The beneficial effects of steroids are achieved through various mechanisms of action. The other options include chemotherapy, radiotherapy, or a combination of both [16, 23]. We surgically resected the intramedullary tumor for possible restoration of neurological function through decompression and for pathological tissue diagnosis. We also used steroids to reduce the neurological deficit. Additionally, in some cases, spinal angiography is performed preoperatively for tumor‐feeding vessel embolization, which can help reduce bleeding during surgery and decrease tumor size [20]. Some studies suggest that performing vascular embolization of the spinal tumor is associated with positive outcomes [17]. Some also suggest that using multimodality therapy, including radiation (systemic or intrathecal) and surgery, is associated with a higher survival compared to single therapy [14]. Some studies demonstrate that surgery prolongs the survival rate [12, 24], while other studies dispute this [14, 25]. Some studies show that concomitant multiple ISCMs are associated with poor survival [12], while other studies find no significant difference in the survival of patients with one or multiple ISCM regions [14]. Based on the literature, metastasis to the spinal cord significantly reduces the quality of life [18]. Additionally, ovarian cancer metastasis to the spinal cord has poor outcomes with low survival (from 10 months to 3 years) and likely depends on the delayed diagnosis from initial symptom onset and therapies received by patients. Thus, better defining this disorder pattern and early identifying metastasis could prevent delayed diagnosis and permanent neurological injury [9, 16, 22]. The prognosis of these patients is highly dependent on their neurological status at the time of admission and after treatment, the velocity of neurological deficit initiation, the type of primary neoplasm, early diagnosis, and the proper method of treatment. Therefore, rapid evaluation of neurological status is crucial, as missing time may impose a significant burden on a patient's morbidity and mortality [9, 19, 24]. Some suggest that the prognosis may be affected by the interval between the diagnosis of the primary cancer and metastasis to the spinal cord. They showed that longer survival after treatment is associated with a lower speed of metastasis [19]. Unfortunately, at the time of admission, we found that the patient has paresthesia, motor, and sensory deficits, which are prognostic factors associated with poor outcome. Additionally, the interval between the primary ovarian adenocarcinoma and ISCM was 36 months.

A comprehensive search of the literature in PubMed, Google Scholar, Web of Science, and Scopus was performed from 2016 to date to determine relevant articles on the intramedullary spinal cord metastases from ovarian carcinoma. To date, there have only been seven cases defined as intramedullary metastasis (without associated cranial and extramedullary metastasis), of which two were in the cervical spine, four in the thoracic spine, and one in the cervical–thoracic spine level. The initial condition in all cases was treated through surgical removal, and systemic chemotherapy was performed for six of them. Just one patient was known to have metastasis at the time of primary diagnosis, while the other six patients were presumed disease free during follow‐up until they progressed neurological deficit. In our literature study, we found that ISCM occurred in a wide spectrum of patients, ranging in age from 56 to 78 years, with an average age at diagnosis of 64.8 years. The typical duration for diagnosing metastasis within the spinal cord was about 17.7 months after the primary diagnosis, while the metastasis timing of one case in the literature review was not available. All patients underwent surgical resection for ISCM, and adjuvant chemotherapy was performed for three cases. Additionally, three cases were treated with radiation therapy as adjunctive therapy. Additionally, four cases underwent steroid therapy. Two patients died (one after 1 year and the other's death time is not available), and the other five individuals continued to remain disease free [Table 1].

**TABLE 1:** Summary of isolated intramedullary metastasis from primary ovarian carcinoma published in literature till date.

Authors	Age (year)	Histology of primary	Level of lesion	Time from primary diagnosis to spinal metastasis diagnosis*	Treatment	Outcome
Safadi et al. (2016) [9]	78	Ovarian adenocarcinoma	T11/T12	17 months	Surgery Chemotherapy Radiotherapy Steroids	Improvement in her lower extremity strength, but she did not regain full function
Soylemez et al. (2017) [21]	56	Poorly differentiated ovarian serous cystadenocarcinoma Grade IIIc	C6/C7	NA	Gross total resection	Disease free at 3 years follow‐up
Multani et al. (2021) [16]	74	Serous ovarian papillary adenocarcinoma. Grade III	D6/D7	18 months	Gross total resection Radiotherapy Steroids	Neurologically improved/disease free till 2 years than lost to follow‐up
Kłębczyk et al. (2021) [26]	66	Serous ovarian cancer	T12	24 months	Gross total resection	Neurologically partially improved
Huang et al. (2017) [27]	50	Ovarian adenocarcinoma	C7–T1	24 months	Microsurgical resection Chemotherapy Steroids	Neurologically improved
Ravindra et al. (2017) [28]	59	Ovarian adenocarcinoma	T10/T11	24 months	Surgery Radiotherapy Steroids	Neurologically improved (but proprioceptive loss in her left foot, and developed bladder incontinence)/died 1 years after
Nikolaidi et al. (2020) [29]	71	Ovarian serous carcinoma	C5–C7	17 months	Surgery Chemotherapy (a single cycle)	Died
Our case	70	Papillary serous ovarian adenocarcinoma	T2/T4	36 months	Gross total resection Steroids	Died (20 days later)

Abbreviation: NA, not available.

* Approximate time in some cases based on estimates provided in reference.

Based on our research findings, we advise that physicians have a notably low threshold when considering diagnosing metastatic CNS lesions in advanced‐stage ovarian carcinoma. Additionally, perform instruments such as neuroaxis MRI for screening in follow‐up visits even with normal levels of CA‐125. It is acceptable to have a variety of management options for patients, while a distinct method based on the patient's condition should be tailored, and this may be different from case to case. The treatment choice should be personalized after discussion with a medical oncologist, neurosurgeon, neuro‐oncologist, and radiation oncologist. The essential goal is decreasing mortality and enhancing the quality of life of patients.

In the above case report, the tissue diagnosis confirmation was obtained. The patient did not show postoperative neurological improvement. Our patient was discharged from the hospital after 7 days and was advised to return about 10 days later. In the follow‐up, we recognized that, unfortunately, 3 weeks after surgery, the patient ultimately passed away due to respiratory and circulatory system failures. Surgical therapy is associated with some complications that may lead to critical conditions and eventually death. For example, embolic events due to diminished activity, may result in cardiopulmonary complications leading to death. Additionally, wound infections, which may be treated with drainage or antibiotic therapy, epidural hematoma, and collection of CSF due to leakage can be removed with drainage. Worsening of each of these complications may lead to morbidity and mortality. Unfortunately, the patient went to another hospital, and her family declined to authorize an autopsy to discern the cause of death.

## 4 Conclusion

For the management of patients with a probable diagnosis of metastatic intramedullary spinal cord tumor with neurological dysfunction, there is no unanimity on definitive strategies. In patients with spinal cord metastasis who experience progression of neurological dysfunction and whose medical conditions are stable enough for surgery, we suggest open surgical biopsy with resection. This approach follows multiple goals: it confirms the diagnosis of tissue, diminishes tumor load for subsequent adjuvant therapies, minimizes surgical morbidity, and aims for definitive diagnosis and treatment of nonmetastatic diseases that may present as intramedullary spinal cord metastases. The benefits and risks of this procedure, however, must be carefully assessed with individual patients and their families. To improve the survival rate and longer life expectancy of cancer patients after their primary diagnosis and treatment, it will be essential for neurosurgical oncologists to work closely with radiation oncologists and medical oncologists to adjust individualized management methods for patients with metastases to the CNS, considering a risk–benefit assessment and taking into account the patient's preference for quality of life and potential survival duration.

## Author Contributions

**Vahid Heidari:** methodology (equal), project administration (equal), resources (equal), supervision (equal), validation (equal), visualization (equal). **Reza Mollahoseini:** methodology (equal), resources (equal). **Navid Golchin:** resources (equal). **Hadi Karimi:** resources (equal). **Mohammad Hoseini:** resources (equal). **Benyamin Kazemi:** project administration (equal), resources (supporting). **Elmira Zolfeghari:** resources (equal), project administration (equal). **Hemin Ashayeri Ahmadabad:** conceptualization (lead), data curation (equal), investigation (lead), project administration (equal), resources (equal), software (lead), supervision (equal), validation (equal), writing – original draft (lead), writing – review and editing (lead).

## Ethics Statement

In accordance with international ethical standards, the author(s) have obtained and securely retained written ethical permission.

## Consent

The patient provided written consent to share their medical information and treatment details in the medical literature as a rare case.

## Conflicts of Interest

The authors declare no conflicts of interest.

## Data Availability

Data sharing is not applicable to this article as no new data were created or analyzed in this study.
